# Synergistic antibacterial activity of *Lactococcus lactis* and *Xylella* phage MATE 2 for an effective biocontrol strategy against black rot disease in broccoli

**DOI:** 10.3389/fmicb.2024.1468792

**Published:** 2024-08-19

**Authors:** Miloud Sabri, Kaoutar El Handi, Abderrahim El Tousy, Angelo De Stradis, Toufic Elbeaino

**Affiliations:** ^1^International Centre for Advanced Mediterranean Agronomic Studies (CIHEAM of Bari), Valenzano, Italy; ^2^National Research Council of Italy (CNR), Institute for Sustainable Plant Protection (IPSP), University of Bari, Bari, Italy; ^3^National Research Council of Italy (CNR), Institute for Sustainable Plant Protection (IPSP), Piazzale Enrico Fermi, Portici, Italy

**Keywords:** phage-lactic acid bacteria synergy, bacteriocin, biocontrol, *Xanthomonas campestris* pv. *campestris*, cruciferous vegetables

## Abstract

Black rot, caused by *Xanthomonas campestris* pv. *campestris* (*Xcc*), is considered the most destructive disease affecting cruciferous vegetables, resulting in significant losses worldwide. The need for biocontrol agents against *Xcc* that can reduce reliance on chemical pesticides, enhance sustainability, and ensure crops and environmental health is crucial. Combining phages with other antibacterial agents (i.e., antibiotics and bacteriocins) to treat bacterial infections is gaining increased attention due to the frequently observed synergistic effects. This study introduces for the first time the combination of a lytic phage, i.e., *Xylella* phage MATE 2 (MATE 2) with nisin-producing *Lactococcus lactis* subsp. *lactis* (*L. lactis*) bacterium as an eco-friendly, cost-effective, and practical strategy for controlling *Xcc* in cruciferous vegetables. The antibacterial efficacy of MATE 2 and *L. lactis*, individually and in combination, against *Xcc* was investigated through a series of *in vitro* assays and *in planta* experiments conducted on broccoli plants. The time-killing curves results showed that under conditions of reduced *Xcc* population concentration (10^3^ CFU/mL), MATE 2 at 10^8^ PFU/mL exerted a persistent inhibitory effect on *Xcc* growth for 7 days. The Spot assays and v-qPCR analysis showed that both *L. lactis* and its bacteriocin nisin have significant antibacterial potential to contrast *Xcc*. Furthermore, combined application of MATE 2 and *L. lactis* in broccoli plants by foliar spraying generated significant synergistic efficacy in preventing *Xcc* infections, achieving a 71% reduction in symptoms, compared with 64 and 38% for single applications, respectively. In this study, the positive synergistic effect of the combined application of phage and beneficial bacteria in preventing black rot disease underscores this eco-friendly and cost-effective approach as a promising control measure against plant bacterial diseases.

## 1 Introduction

*Xanthomonas campestris* pv. *campestris* (*Xcc*) is a Gram-negative bacterium belonging to the family *Xanthomonadaceae*, which includes the most devastating plant pathogens that continually threaten food security (Dimkić et al., 2023). *Xcc* is an economically important phytopathogen that causes black rot disease in many *Brassicaceae* species (Alvarez, 2000; Papaianni et al., 2020). Black rot is considered the main yield-limiting and destructive disease of brassica vegetable crops worldwide, including cabbage, radish, cauliflower, broccoli, kale, and Brussels sprouts (Vicente and Holub, 2013; Hakalová et al., 2022). Characteristic symptoms of black rot are V-shaped yellow lesions starting from the leaf margins and blackening of the veins that results from *Xcc* movement in the vascular system (Vega-Álvarez et al., 2021). The affected leaves can fall prematurely, and the disease can cause stunted growth and the death of young plants, significantly affecting crop biomass and quality (Vicente and Holub, 2013). The pathogen can live epiphytically on leaf surfaces or as a saprophyte in necrotic tissues and generally enters the plant through hydathodes on leaf margins or through wounds and colonizes the plant’s vascular system (Danhorn and Fuqua, 2007; Boulanger et al., 2014).

Historically, the control of *Xcc* has been challenging and primarily relies on the use of *Xcc*-free planting material (seeds or transplants), crop rotation, and the elimination of other potential inoculum sources such as residues of infected crops and cruciferous weeds (Iglesias-Bernabé et al., 2019). The development and use of resistant cultivars has had limited success due to the scarcity of resistance sources and the existence of nine races of the pathogen (Vicente and Holub, 2013; Iglesias-Bernabé et al., 2019). Additionally, chemical treatments and the use of antibiotics in crop production are being hampered by the emergence of resistant bacterial strains, soil accumulation, as well as growing concerns about their environmental and health implications (Behlau et al., 2020; Holtappels et al., 2022). Nowadays, bacteriophages (viruses that specifically infect and lyse bacteria) are being evaluated as alternative biocontrol agents to be integrated into the management of plant diseases (Anyaegbunam et al., 2022). Lytic bacteriophages are responsible for the destruction of the host bacterial population by infecting and replicating within the bacteria, causing lysis and release of new phage particles, which can then infect other bacterial cells (Greer et al., 2024). In the case of black rot disease, numerous studies have highlighted the potential usefulness of implementing phage therapy to combat *Xcc* infections (Papaianni et al., 2020; Holtappels et al., 2022).

Phage therapy is currently one of the recommended therapeutic options, preferably in combination with another antibacterial agent, a practice thought to improve efficacy (Bhargava et al., 2021). Several studies have reported the potential of bacteriophages to act synergistically with other antimicrobial agents such as antibiotics and bacteriocins, reinforcing the concept that when combined, they are more efficient (Gu Liu et al., 2020; Bhargava et al., 2021; Rendueles et al., 2022). Among antibacterial agents, *Lactococcus lactis* subsp. *lactis* (*L. lactis*) represents one of the most extensively studied lactic acid bacteria (LAB) due to its aptitude to produce nisin, a bacteriocin that exhibits potent antibacterial activity against both Gram-positive and Gram-negative disease-associated pathogens (Shin et al., 2016; Bukvicki et al., 2020). This LAB and its cell-free supernatant have been shown to exhibit antibacterial activity against *Xanthomonas* spp. strains (Mačionienė et al., 2022). Additionally, Serna-Cock et al. (2013) had also showed that *L. lactis* exerts an *in vitro* antimicrobial activity against *Xanthomonas albilineans*. Nevertheless, research on the efficacy of *L. lactis* against *Xcc* infections is still lacking.

Nisin, recognized as an FDA-approved bacteriocin, is widely used in food preservation and veterinary medicine for the treatment of dermatological conditions and intramammary infections in animals (Greer et al., 2024). It exerts an antibacterial activity by generating pores in the cell membrane and interrupting cell wall biosynthesis through specific lipid II interaction (Prince et al., 2016). In the field of plant protection, nisin has been demonstrated to be effective *in vitro* and *in planta* against plant pathogenic bacteria belonging to the *Xanthomonadaceae* family, namely *Xylella fastidiosa* (*Xf*) (Sabri et al., 2024a). Additionally, the combination of nisin with lytic phages has demonstrated high potential to simultaneously control *Salmonella typhimurium*, *Salmonella enteritidis*, and *Escherichia coli* (Duc et al., 2023). This encourages the exploration of the potential use of *L. lactis* in combination with *Xcc* lytic phages as a cost-efficient biocontrol strategy for combating *Xcc* infections *in planta*. Accordingly, this study offers a viable alternative to conventional pesticides by integrating the *Xylella* phage MATE 2 (Sabri et al., 2024b) with *L. lactis* and explores for the first time their antibacterial activities against *Xcc in vitro* and *in planta*.

## 2 Materials and methods

### 2.1 Bacteriophage, bacterial strains, and culture conditions

*Xcc* strain CFBP 1710 and *L. lactis strain* ATCC 11454 were grown either in liquid yeast extract peptone glucose broth (YPG) (5.0 g/L yeast extract, 5.0 g/L peptone and 10.0 g/L glucose) or on yeast extract peptone glucose agar (YPGA, i.e., YPG supplemented with 1.5% agar). The strains were stored at −80°C in 25% glycerol in YPG broth. Before use, the strains were plated from glycerol stocks onto YPGA agar plates and incubated at 28°C for 24 h. The bacteriophage named *Xylella* phage MATE 2 (MATE 2) from our collection, which possessed a broad spectrum of antibacterial activity against various phytobacteria of the *Xanthomonadaceae* family (Sabri et al., 2024b), including *Xcc*, was used in this study.

Nisin (ITSISLCTPGCKTGALMGCNMKTATCHCSIHVSK), extracted and purified from *L. lactis*, was purchased from Sigma-Aldrich (Merck KGaA, Darmstadt, Germany). According to the manufacturer, the formulation contains 2.5% (w/w) pure nisin with potency ≥900 IU/mg. Lyophilized nisin was solubilized in sterile Milli-Q water to a stock concentration of 12.5 mg/mL and filter sterilized through a 0.22 μm nylon Acrodisc^®^ syringe filter (Merck, Rome, Italy).

### 2.2 Spot assay

The lytic activity of MATE 2 against *Xcc* was assessed using a spot assay as follows: 200 μL of *Xcc* suspension (OD_600_ = 0.2) were mixed with 6 mL of YPG soft agar (i.e., YPG supplemented with 0.7% agar), poured into YPGA plates, and allowed to dry. Subsequently, drops of 10 μL of phage solution at 10^8^, 10^7^, 10^6^, 10^5^, 10^4^, 10^3^, 10^2^, and 10 PFU/mL were spotted onto the surface of the plates. Spots were dried at room temperature and the plates cultured for 24 h at 28°C.

### 2.3 MATE 2 inhibitory effect on *Xcc* growth

To evaluate the lysis potential of MATE 2 in suppressing the growth of *Xcc* in liquid culture over a 7-day growth period, a bacterial growth reduction assay was performed. Briefly, 100 μL of MATE 2 suspensions (10^8^ and 10^4^ PFU/mL) were separately mixed with 100 μL of each concentration of *Xcc* (ranging from 10^8^ to 10^2^ CFU/mL) in triplicates. The mixture was then inoculated into 2 mL of YPG broth in a 10 mL centrifuge tube and incubated at 28°C for 7 days. During incubation, 11 optical density measures (0, 4, 8, 18, 24, 48, 72, 96, 120, 144, and 168 h) at OD_600_ were obtained using the UV spectrophotometer (UV -1800, Shimadzu Corporation, Kyoto, Japan). All the data are presented as mean ± standard deviation (SD) of three independent experiments.

### 2.4 *In-vitro* investigation for antagonism between *Lactococcus lactis* and *Xcc*

The antagonistic potential of *L. lactis* ATCC 11454 against *Xcc* was assessed through a spot assay as follows: 300 μL of *Xcc* suspension (OD_600_ = 0.1) were equally spread on YPGA plates and allowed to dry under the laminar flow hood. Subsequently, drops of 10 μL of *L. lactis* suspension (OD_600_ = 0.2) were spotted onto the surface of the plates. Spots were dried at room temperature and the plates were incubated for up to 2 days at 28°C. Sterile distilled water served as the negative control. The antagonistic activity of *L. lactis* was perceived as an inhibition zone of *Xcc* growth, which was measured using a digital caliper. This experiment was conducted in triplicates.

### 2.5 Antibacterial activity of nisin against *Xcc*

*L. lactis* ATCC 11454 is well known as one of the main nisin producers (Penna et al., 2005). In this regard, viable-quantitative PCR (v-qPCR), a suitable method for estimating the viability of bacterial cells (Baró et al., 2020), and spot assay were performed to explore whether nisin was involved in the antagonistic activity exhibited by *L. lactis* ATCC 11454 against *Xcc*. For v-qPCR assay, 50 μL of *Xcc* suspension (OD_600_ = 0.2) were treated with 50 μL of nisin at 6, 4, 3, 2, 1, 0.8, 0.6, 0.4, 0.2, and 0.1 mg/mL and incubated for 3 h at 28°C. Viable and dead *Xcc* cells (OD_600_ = 0.2) treated at 95°C for 15 min were served as controls. After incubation, samples were treated with PMAxx^™^ (Biotium, Rome, Italy) at a final concentration of 7.5 μM, incubated in the dark at room temperature for 8 min, and followed by photoactivation for 15 min using the PMA-Lite™ LED Photolysis Device (Biotium, Fremont, United States). Genomic DNA of all samples was extracted following the cetyltrimethyl ammonium bromide (CTAB) protocol (Wilson, 2001). V-qPCR was carried out in a thermocycler apparatus (Bio-Rad CFX96, BioRad, Milan, Italy), using the primers HrcCF2 (5′-CGTGTGGATGTGCAGACC-3′) and HrcCR2 (5′-CAGATCTGTCTGATCGGTGTCG-3′) (Papaianni et al., 2020). For spot assay, 300 μL of *Xcc* suspension (OD_600_ = 0.2) were equally spread on YPGA plates and allowed to dry under the laminar flow hood. Subsequently, drops of 10 μL of nisin at 6, 4, 3, 2, 1, 0.8, 0.6, 0.4, 0.2, and 0.1 mg/mL were spotted onto the surface of the plates as described earlier.

### 2.6 Fluorescence microscopy

To further evaluate the bactericidal activity of nisin against *Xcc* cells, 100 μL of *Xcc* suspension (OD600 = 0.2) was mixed with 100 μL of nisin at 2 mg/mL and incubated at room temperature for 3 h. The LIVE/DEAD^®^ BacLight^™^ viability kit (ThermoFisher Scientific, Milan, Italy) was used according to the manufacturer’s instructions to assess the viability of *Xcc* cells treated with nisin at 15 min, 30 min, 1 h, 2 h and 3 h post-incubation. Photomicrographs were taken using a Nikon E800 microscope equipped with fluorescein isothiocyanate (480/30 excitation filter, DM505 dichroic mirror, 535/40 emission filter) and tetramethyl rhodamine isothiocyanate (546/10 excitation filter, DM575 dichroic mirror, 590 emission filter) fluorescence filter sets.

### 2.7 Transmission electron microscopy

The antibacterial effect of nisin against *Xcc* cells at the ultrastructural level was assessed using a TEM. Briefly, 100 μL of *Xcc* suspension (OD600 = 0.2) was treated with 100 μL of nisin at 2 mg/mL for 3 h at room temperature. Micrographs were taken after 3 h via TEM (FEI MORGAGNI 282D, United States) using the dip method. Briefly, carbon-coated copper/rhodium grids were immersed in both the nisin-treated and untreated bacterial suspensions for 5 min, followed by a rinse with 200 μL of distilled water. To achieve negative staining, the grids were floated on 200 μL of a 0.5% w/v UA-zero EM stain solution (Agar-Scientific Ltd., Stansted, United Kingdom), and observed under an accelerating voltage of 80 kV.

### 2.8 Nisin-MATE 2 combination against *Xcc*

To study the synergistic effects, 50 μL of phage suspension (10^8^ PFU/mL) was added to 50 μL of nisin at the sublethal concentration (1 mg/mL) and mixed with 100 μL of *Xcc* suspension (OD600 = 0.2). Additionally, 100 μL of MATE 2 (10^8^ PFU/mL) and 100 μL of nisin (1 mg/mL) were individually mixed with 100 μL of *Xcc* suspension (OD600 = 0.2) and served as controls. The mixtures were then inoculated into 2 mL of YPG broth in a 10 mL centrifuge tube and incubated at 28°C for 3 days. Optical density measures were determined using the UV spectrophotometer and compared to the controls.

### 2.9 *In planta* antagonistic effects of *Lactococcus lactis* and MATE 2 against *Xcc*

To evaluate the efficacy of *L. lactis* and MATE 2, individually and in combination, in contrasting *Xcc* infections within plant tissues, an *in-planta* assay was carried out on one-month-old broccoli plants (*Brassica oleracea* var. *italica*) in a greenhouse under controlled conditions (25 ± 2°C and ~ 70% humidity). Three leaves per plant were wounded by multi-needle prickers and then sprayed with the *Xcc* suspension (0.66 mL/plant; OD600 = 0.2) and the biocontrol agents. *L. lactis* (2.5 mL/plant; OD600 = 0.7) and MATE 2 (2.5 mL/plant; 10^8^ PFU/mL), individually and in combination, were applied preventively, i.e., 24 h prior to *Xcc* inoculation, and curatively (24 h after *Xcc* inoculation). Ten broccoli plants were used for each treatment. Untreated plants inoculated with sterile water or with *Xcc* only were included as negative and positive controls, respectively. Fourteen days post-infection (dpi), disease symptoms were quantified by measuring the area of necrosis on the treated leaf according to an arbitrary six-level disease scale whereby: 0 = no symptoms, 1 = symptoms begin to appear, 2 = ¼ of leaf area showing symptoms, 3 = ⅓ of leaf area showing symptoms, 4 = ½ or more of leaf area showing symptoms, 5 = the entire leaf showing symptoms. The efficacy of each treatment in reducing *Xcc* infections was calculated as follows:


$$\mathrm{Efficacy}\;\left(\%\right)=100-\left(\frac{\sum \left(P\times T\right)}{\sum \left(P\times C\right)}\times 100\right)$$

Where P = severity score, T = number of treated leaves having the same score, and C = number of infected leaves (positive control) having the same score.

### 2.10 Statistical data analysis

To test the antagonistic effect of *L. lactis* and MATE 2 on *Xcc* infections in broccoli plants, a one-way analysis of variance (ANOVA) was performed using the IBM SPSS Statistics 26 software. The significance of differences was calculated by Duncan’s *post-hoc* test and a *p*-value less than 0.05 was considered statistically significant. All data are presented as mean ± standard deviation (SD) of two independent experiments.

### 2.11 Persistence of *Lactococcus lactis* and MATE 2 in broccoli plants

To investigate the persistence of *L. lactis* and MATE 2 within broccoli plants, the treated leaves were analyzed at the end of the *in-planta* experiment (14 dpi). Briefly, two leaves from each treatment were ground in 4 mL of sterile water and centrifuged at 1,000 g for 5 min to remove plant debris. In the case of *L. lactis* samples, the resultant supernatant was diluted six times using a 10-fold serial dilution and 50 μL were plated on YPGA plates. The plates were incubated at 28°C for 24 h and *L. lactis*-like colonies were purified on YPGA plates and subjected to PCR testing using the *L. lactis* subsp. *lactis*-specific primers Nis-F: 5′-CGGCTCTGATTAAATTCTGAAG-3′ and Nis-R: 5′-GGATTAGCTAGTAGTAACTGTTC-3′ reported in Alegría et al. (2010). For MATE 2 samples, the macerate was filtered through 0.22-μm filter and 500 μL of the filtrate was added to 200 μL of *Xcc* suspension (OD600 = 0.2). The mixture was then inoculated into 2 mL of YPG broth in a 10 mL centrifuge tube and incubated for 24 h at 28°C for phage enrichment. The phage was then tested against *Xcc* using a spot assay and subjected to PCR using MATE 2-specific primers MATE 2-F: 5′-CCCGCCCAGGATCGCAAGCCGT-3′ and MATE 2-R: 5′-TCGTGCTCTCAGATTGTTGACGC-3′ designed in this study. The PCR cycles consisted of an initial denaturation step at 94°C for 5 min; 35 cycles of 94°C for 30 s, 60°C for 30 s, and 72°C for 30 s; and a final elongation step at 72°C for 5 min.

## 3 Results

### 3.1 Spot assay

**Figure 1:**
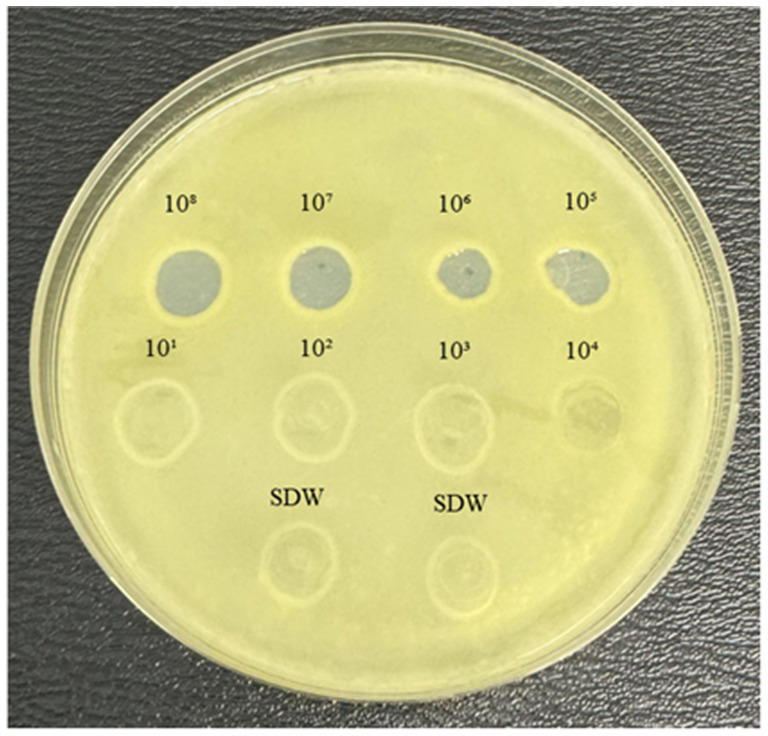
YPGA agar plate showing the lytic activity of MATE 2 at different titers (10^8^–10^1^ PFU/mL) against *Xcc*. SDW: sterile distilled water used as a negative control.

The lytic activity of MATE 2 against *Xcc* was initially examined through a spot assay. The results showed that MATE 2 produced clear lysis zones on the *Xcc* lawn at 10^8^, 10^7^, 10^6^, and 10^5^ PFU/mL (Figure 1), indicating complete lysis of *Xcc* bacteria. At 10^4^ PFU/mL, the lysis zone turned semi-translucent, demonstrating reduced antibacterial activity. Beyond this titer, no distinct lysis zones were observed, indicating that the phage titer was insufficient to induce distinct effect on *Xcc* lawn. These results highlight the lytic effectiveness of MATE 2 against *Xcc* and shed light on phage concentration requirements for combating *Xcc* infections.

### 3.2 Bacteriolytic effect of MATE 2 on *Xcc* growth

**Figure 2:**
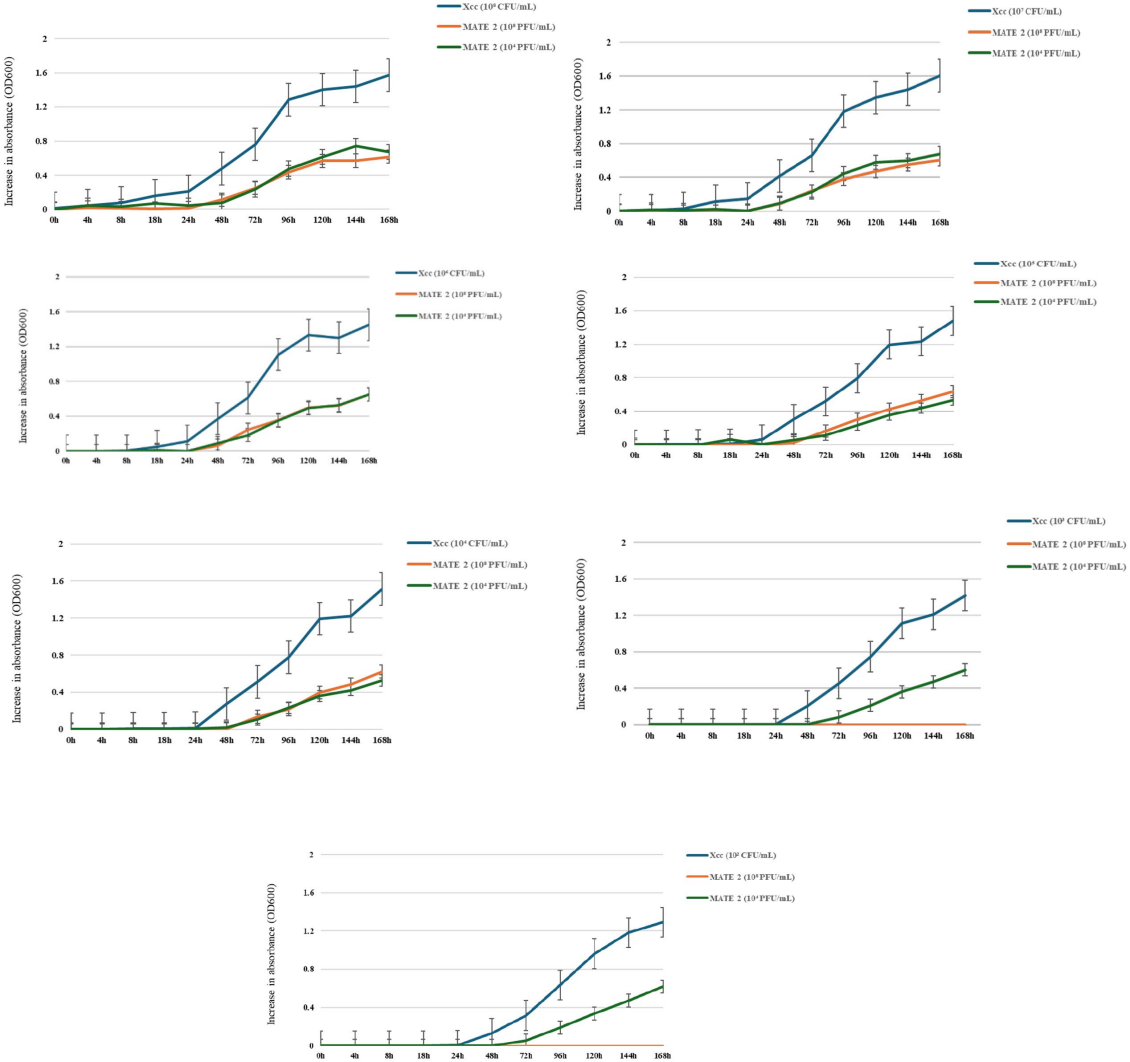
Time-killing curves showing the bacteriolytic effect of MATE 2 at 10^8^ and 10^4^ PFU/mL on *Xcc* growth at various concentrations (10^8^, 10^7^, 10^6^, 10^5^, 10^4^, 10^3^, and 10^2^ CFU/mL). The optical density of treated and untreated-bacterial culture with MATE 2 at 24-h interval for 7 days is compared. The bars indicate the error means for three replicates.

To examine the dynamics of MATE 2 virulence, killing curves were generated by infecting *Xcc* at concentrations of 10^8^, 10^7^, 10^6^, 10^5^, 10^4^, 10^3^, and 10^2^ CFU/mL with MATE 2 at two titers of 10^8^ and 10^4^ PFU/mL. These latter were selected based on the results of the spot assay, wherein a titer of 10^4^ PFU/mL was identified as the minimum titer of MATE 2 capable of inducing a lytic effect on *Xcc* lawn. The results showed that both phage titers effectively inhibited the growth of all *Xcc* cultures for 24 h (Figure 2). However, after 24 h, MATE 2-infected bacteria at high concentrations (10^8^, 10^7^, and 10^6^ CFU/mL) started to display an increase in OD reading, indicating the emergence of phage resistance (Figure 2). Despite the resistance development, MATE 2 showed significant antibacterial activity against *Xcc* growth, with an inhibition rate of 60%, 7 dpi. *Xcc* at 10^5^ and 10^4^ CFU/mL concentrations developed resistance to both phage titers after 48 h, similar to *Xcc* concentrations (10^3^ and 10^2^ CFU/mL) treated with MATE 2 at 10^4^ PFU/mL (Figure 2). Most importantly, the infection of *Xcc* at 10^3^ and 10^2^ CFU/mL with MATE 2 at 10^8^ PFU/mL resulted in a persisting inhibitory effect for 7 days, leading to the complete inhibition of *Xcc* growth (OD600 = 0) (Figure 2). At the end of the experiments, MATE 2-resistant mutants were isolated on YPGA agar plates and resistance was confirmed by spot assay. These results provide insights into the *Xcc*-MATE 2 interaction, revealing that a 10^8^ PFU/mL titer appears to be suitable for phage preparation to reduce the development of bacterial resistance. The results also indicated that at controversial concentrations, i.e., low *Xcc* population and high MATE 2 titer, *Xcc* is unable to develop resistance to MATE 2 during 7 days of co-incubation. This suggests that application of MATE 2 at high concentrations could significantly contrast *Xcc* infections and delay the emergence of phage resistance.

### 3.3 Antibacterial activities of *Lactococcus lactis* and nisin against *Xcc*

**Figure 3:**
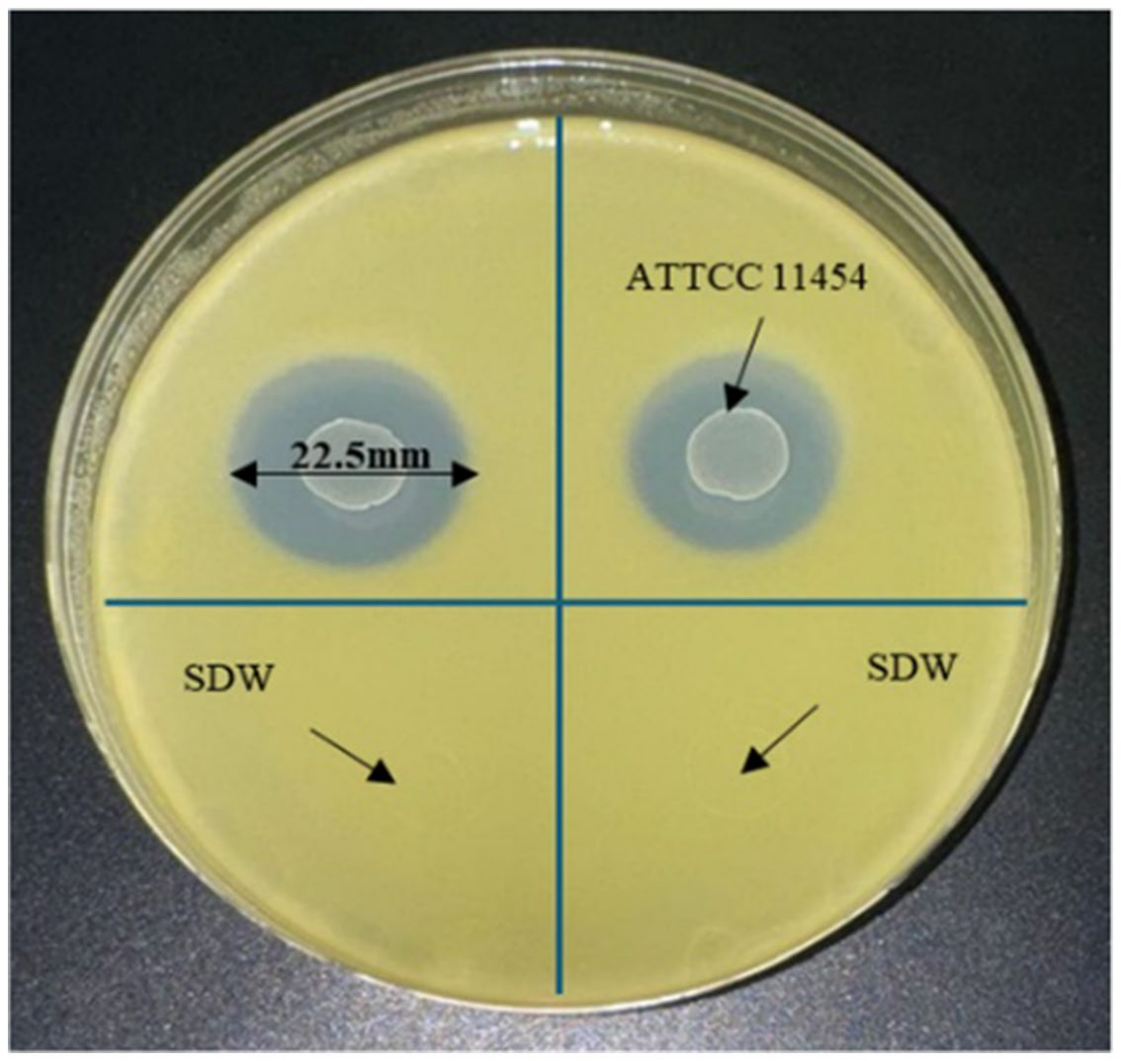
YPGA plate showing the antagonistic effect of *L. lactis* on *Xcc.*

The antagonistic activity of *L. lactis* on *Xcc* cells was examined through *in-vitro* culture assay, resulting in an inhibition zone diameter of 22.5 mm for *Xcc* growth (Figure 3). This inhibitory effect highlights the efficacy of *L. lactis* in contrasting *Xcc*, suggesting its potential use as a biocontrol agent or a source of antimicrobial compounds targeting *Xcc* in agricultural applications.

**Figure 4:**
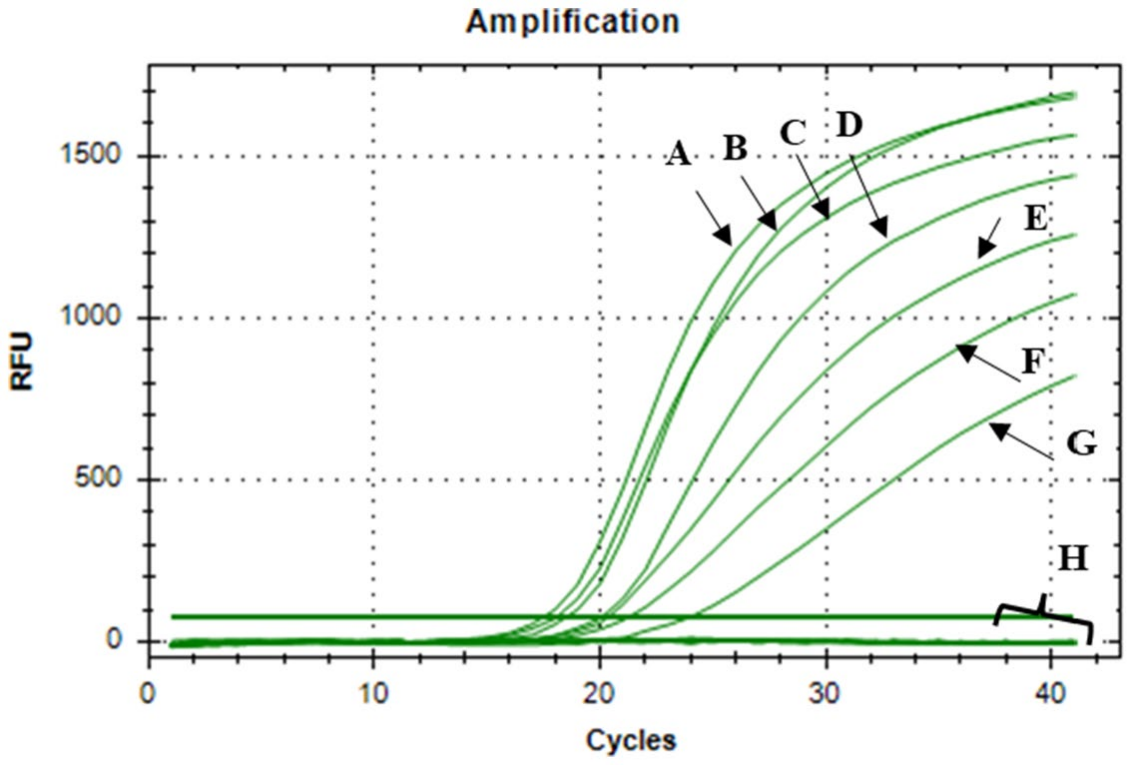
V-qPCR assay showing DNA amplification curves obtained from: (A) untreated *Xcc*, (B) nisin-treated *Xcc* at 0.1 mg/mL, (C) at 0.2 mg/mL, (D) at 0.4 mg/mL, (E) at 0.6 mg/mL, (F) at 0.8 mg/mL, (G) at 1 mg/ mL, (H) DNA amplification curves obtained from nisin-treated *Xcc* at 2, 3, 4, 6 mg/mL, dead *Xcc* cells, and sterile distilled water used as negative control.

**Figure 5:**
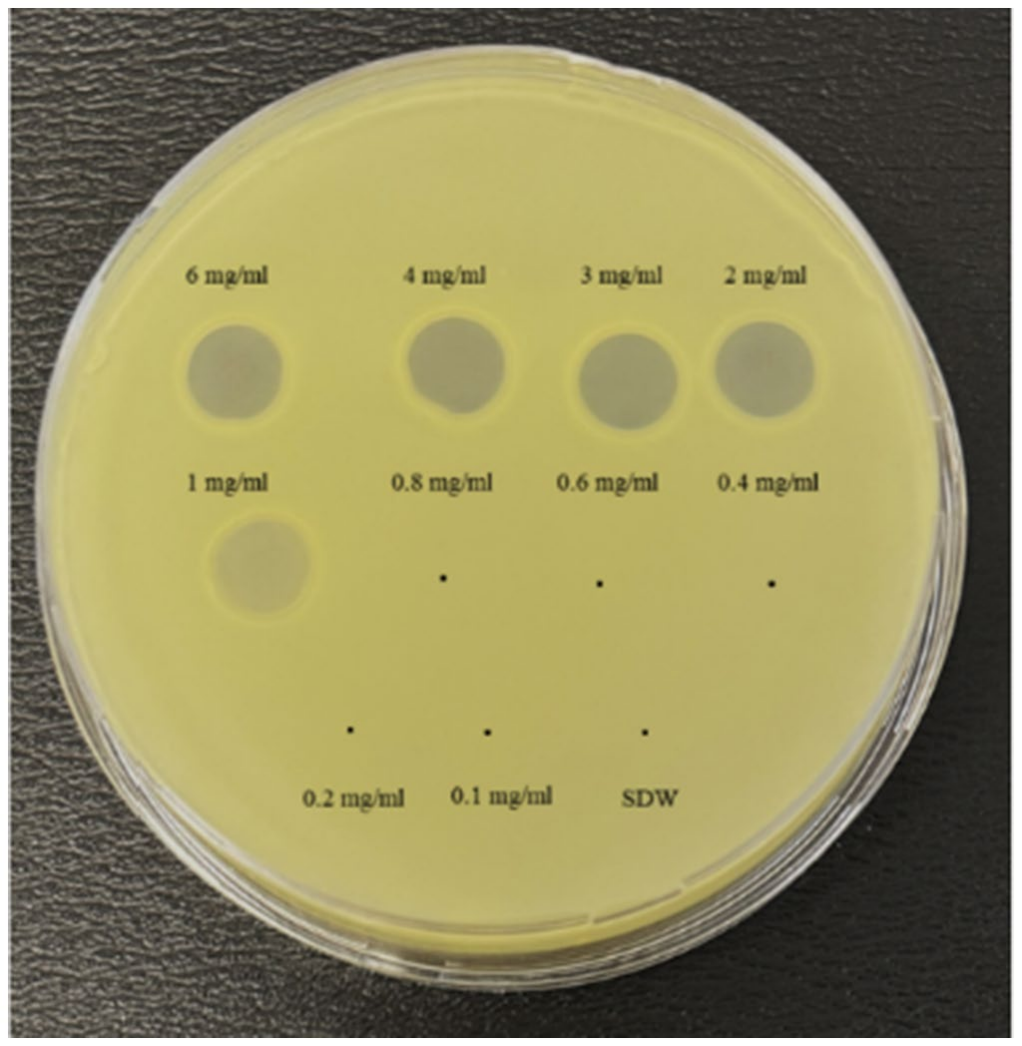
Spot assay showing the antibacterial activity of nisin at various concentrations against *Xcc*. SDW: sterile distilled water used as a negative control.

The bactericidal activity of nisin against *Xcc* was also determined using v-qPCR and spot assays. Results of the v-qPCR showed that nisin exerted antibacterial activity against *Xcc* in a dose-dependent manner (Figure 4). *Xcc* bacteria treated with nisin at 6, 4, 3, and 2 mg/mL exhibited no amplification, similarly to dead *Xcc* cells used as control (Figure 4), indicating complete bacterial lysis. Below these concentrations, nisin showed reduced activity against *Xcc*, with 1 mg/mL as the sublethal concentration. These results were consistent with the spot assay results, which showed clear lysis zones on the *Xcc* lawn induced by nisin at 6, 4, 3, and 2 mg/mL, while 1 mg/mL produced a clouded lysis zone (Figure 5). These findings provide valuable insight into the bactericidal activity of nisin against *Xcc* and suggest that nisin is involved in the antagonistic activity exhibited by *L. lactis* against *Xcc*.

### 3.4 Nisin activity against *Xcc* cells under electron and fluorescence microscopy

**Figure 6:**
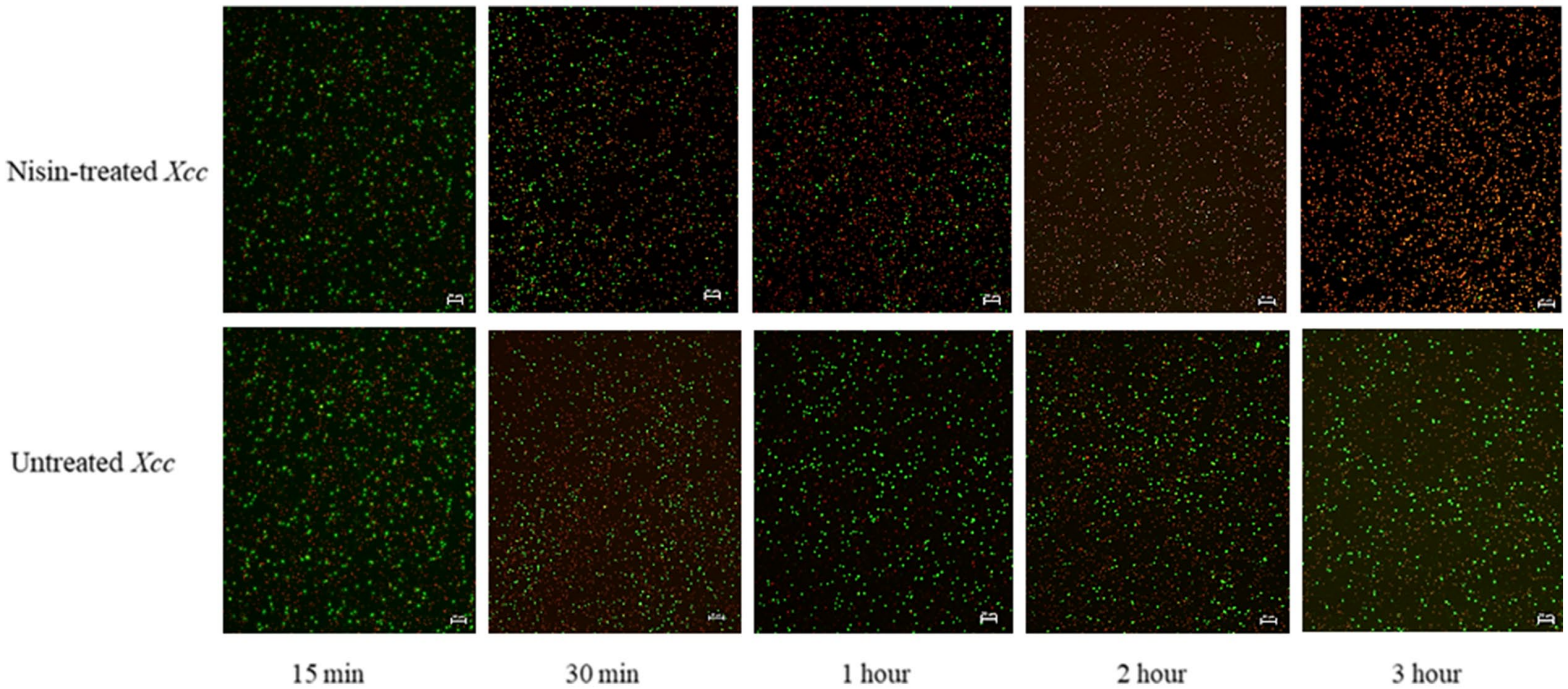
Fluorescent micrographs showing nisin-treated *Xcc* cells at 2 mg/mL. Green and red fluorescence represent live and dead cells, respectively. Magnification 20X, bar 20 μm.

**Figure 7:**
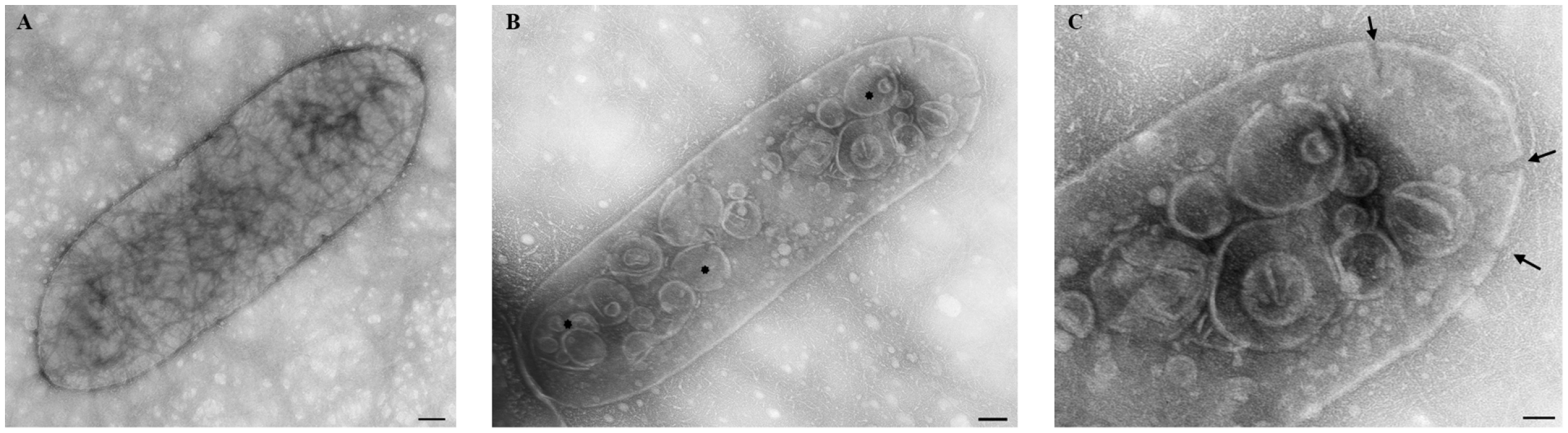
Transmission electron micrographs showing the bactericidal effect of nisin at 2 mg/mL on *Xcc* cells. **(A)** untreated *Xcc* cells, used as control. **(B,C)** Nisin-treated *Xcc* cells showing structural, cell wall, and cytoplasm alterations. Bar **(A,B)** 100 nm; **(C)** 50 nm.

FM and TEM analyses were performed to visually evaluate the bactericidal activity of nisin against *Xcc*. FM micrographs showed that the staining of nisin-treated *Xcc* cells at 2 mg/mL shifted progressively from green (live bacterial cells) to red (dead bacterial cells), indicating damages in the cell membrane (Figure 6). Three hours after contact, intense red channels were observed (Figure 6), indicating robust lysis of *Xcc* and confirming the spot assay and v-qPCR results. At the ultrastructural level, TEM analyses revealed that nisin treatment causes significant structural changes in *Xcc* cells compared to untreated cells (Figure 7A). Electron microscope analysis highlighted the disrupting action of nisin on the bacterial double membrane (Figure 7C, arrows) and the cytoplasm alterations through the production of vesicles (Figure 7B, asterisks), which impair bacterial viability.

### 3.5 Combined effect of nisin and MATE 2 against *Xcc*

**Figure 8:**
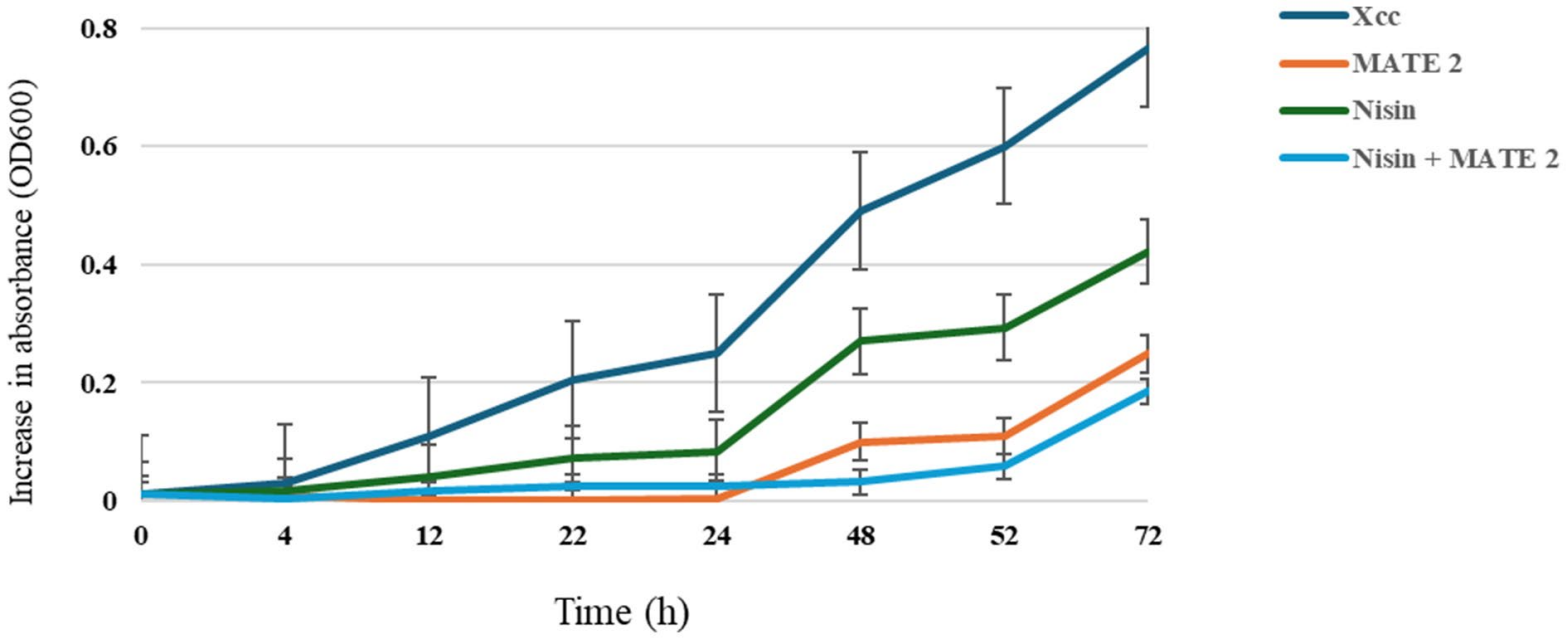
*In-vitro* killing curves displaying the antibacterial activity of MATE 2 and nisin, individually and in combination, against *Xcc.* The bars show the average for three replicates.

The combined effect of nisin and MATE 2 on *Xcc* growth was determined by measuring variations in optical density at OD_600_ over a 3-day incubation period. The results showed that the nisin alone at its sublethal concentration (1 mg/mL) was less effective against *Xcc* growth than MATE 2, resulting in a 45% reduction in *Xcc* growth after 72 h of treatment (Figure 8). Additionally, as previously demonstrated, MATE 2 alone maintained high inhibitory efficacy against *Xcc* for 24 h, after which bacterial growth slightly resumed, resulting in an inhibition rate of 67% after 72 h of co-incubation (Figure 8). Remarkably, the combination of MATE 2 with nisin was more effective against *Xcc* than single treatments, achieving a 76% reduction in bacterial growth (Figure 8). This suggested a synergistic inhibitory effect against *Xcc* and the potential use of MATE 2 and nisin-producing *L. lactis* bacteria to cost-efficiently tackle *Xcc* infections in cruciferous vegetables.

### 3.6 Efficiency of *Lactococcus lactis* and MATE 2 as individual and combined applications in controlling *Xcc* infections in broccoli

**Figure 9:**
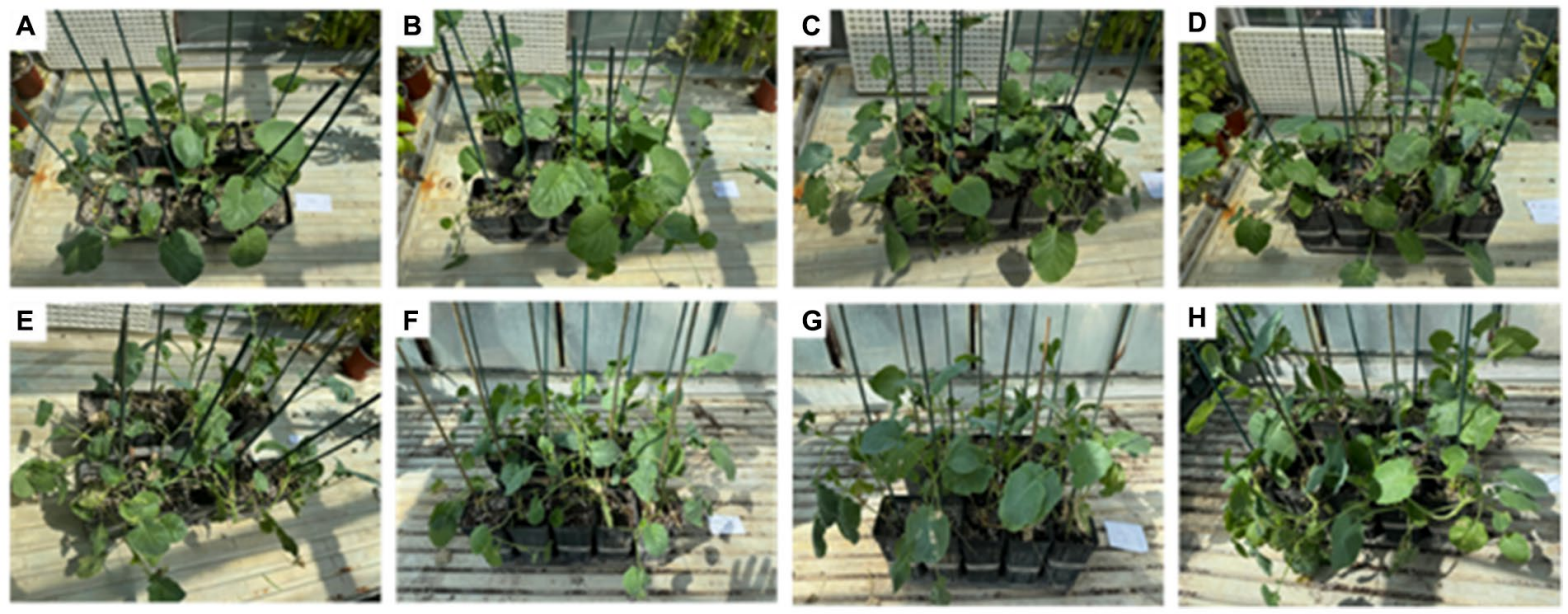
*In-planta* assays showing the antibacterial effects of *L. lactis* and MATE 2, individually and in combination, against *Xcc* infections in broccoli plants 14 dpi. **(A)** Healthy plants used as negative control; **(B)** preventive MATE 2-treated *Xcc*-infected plants; **(C)** preventive *L. lactis*-treated *Xcc*-infected plants; **(D)** preventive MATE 2 + *L. lactis*-treated *Xcc*-infected plants; **(E)** untreated *Xcc*-infected plants used as positive control; **(F)** curative MATE 2-treated *Xcc*-infected plants; **(G)** curative *L. lactis*-treated *Xcc*-infected plants; **(H)** curative MATE 2 + *L. lactis*-treated *Xcc*-infected plants.

**Figure 10:**
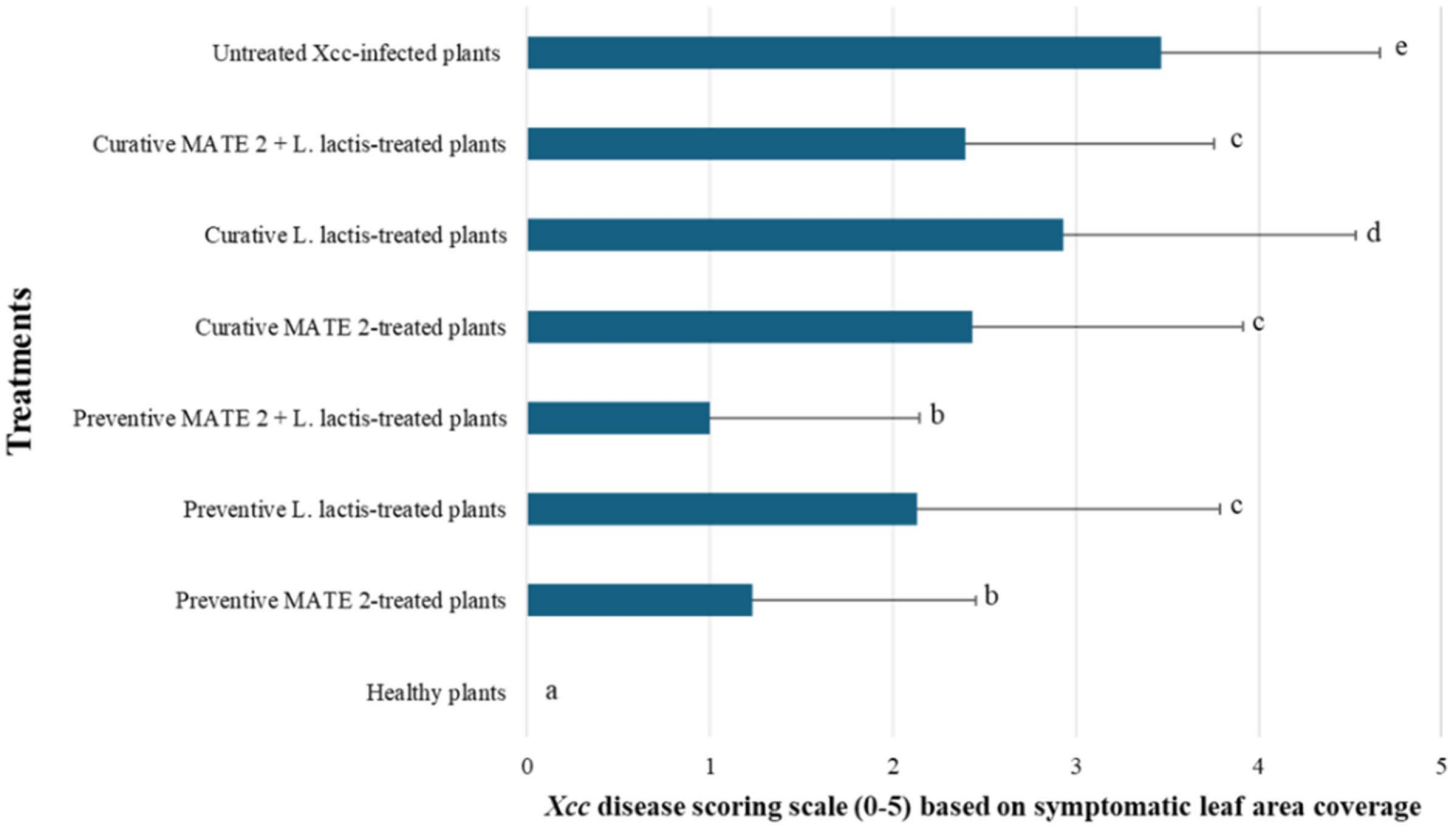
Histograms showing the severity of *Xcc* symptoms on broccoli plants treated preventively and curatively with MATE 2 and *L. lactis*, individually and in combination.

**Figure 11:**
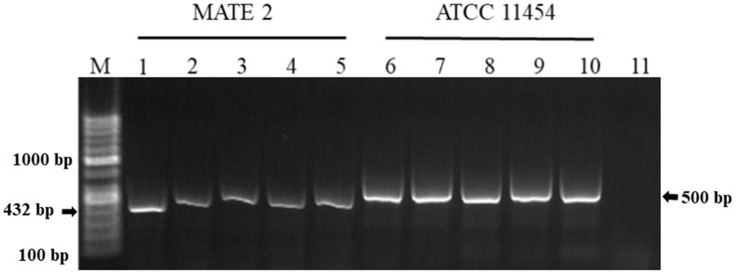
Agarose gel electrophoresis showing PCR products amplified from genomic DNA of *L. lactis* ATCC 11454 and MATE 2 reisolated from broccoli plants 14 dpi. Lane M: 100 bp DNA ladder. Lanes 1–4: MATE 2 reisolated from broccoli plants. Lane 5: MATE 2 used as positive control. Lanes 6–9: *L. lactis* ATCC 11454 reisolated from broccoli plants. Lane 10: *L. lactis* ATCC 11454 used as positive control. Lane 11: sterile water used as negative control reaction.

The *in-planta* antibacterial potentials of MATE 2 and *L. lactis*, individually and in combination, against *Xcc* were evaluated in broccoli plants. The untreated *Xcc*-infected plants (positive control) displayed severe infection symptoms 14 dpi, namely V-shaped yellow lesions on leaf margins, chlorosis, blackening of veins, and loss of leaves, while uninfected plants (negative control) showed no symptoms of *Xcc* (Figure 9). Interestingly, plants treated with MATE 2 and *L. lactis*, individually and in combination, showed reduced *Xcc* symptoms compared to the positive control (Figure 9). In terms of the efficacy of each treatment in reducing *Xcc* symptoms, which was calculated based on the symptom severity scores as described in section 2.9, analysis of our data revealed a very highly significant difference between the treatments (*p* = 0.000) (Figure 10). Once applied preventively, the combination of MATE 2 and *L. lactis* significantly decreased disease symptoms on broccoli leaves by 71% 14 dpi, while their individual applications reduced disease by 64 and 38%, respectively. Curatively, MATE 2 and *L. lactis* exhibited modest efficacy in mitigating *Xcc* symptoms in broccoli plants, achieving respective efficacy rates of 30 and 15%, while their combination resulted in a 31% disease reduction (Figure 10). At the end of the experiment, MATE 2 and *L. lactis* were successfully reisolated from treated broccoli plants and their identity was confirmed by PCR analyses (Figure 11). Furthermore, their persistent antibacterial activity against *Xcc* was validated afterward by spot assays (data not shown). These results clearly indicated the suitability of MATE 2 and *L. lactis* for controlling *Xcc* in cruciferous vegetables and demonstrated their effective combined application in preventing black rot disease.

## 4 Discussion

In agriculture, the use of phages and LAB offers significant advantages, characterized by cost-effectiveness, sustainability, and ease of application (Costa et al., 2023; Ojo and de Smidt, 2023). Phages provide targeted antimicrobial activity against specific bacterial pathogens, thereby lowering the need for broad-spectrum antimicrobials and limiting environmental impacts (Strathdee et al., 2023). Their capacity to multiply inside bacterial hosts enables self-renewal and scalability, adding to cost efficiency in production and application (Ling et al., 2022). Furthermore, phages can be applied in diverse forms, including sprays, drenching, seed treatment and irrigation, facilitating their adoption in agricultural settings (Holtappels et al., 2021). LAB, known for their safety use and production of bacteriocins, offer natural antibacterial properties that improve food preservation and agricultural safety (Ayivi et al., 2020). Their ease of application through fermentation processes and incorporation into probiotic formulations supports practical integration into food production (Timothy et al., 2021). Together, phages and LAB provide cost-effective solutions that support sustainable practices by decreasing the use of chemicals, improving food quality, and managing risks related to pathogens; thus, promoting and building resilience in agricultural and food sectors. To this end, this study reports for the first time the successful use of phage MATE 2 and *L. lactis* in combination to prevent black rot in broccoli plants.

The dynamic of MATE 2 virulence indicated that a high titer of this phage is more efficacious against *Xcc*, maintaining a persistent inhibitory effect on low bacterial populations for 7 days. Similarly, in our previous study we found that MATE 2 was able to significantly inhibit the growth of *Xf* over 7 days with a titer of 10^8^ PFU/mL (Sabri et al., 2024b). However, the emergence of bacterial resistance is a common challenge in phage therapy that is conditioned by many aspects, i.e., phage and bacterial concentrations, lysis potential, etc. Therefore, employing MATE 2 in conjunction with other antimicrobial compounds (bacteriocins) would help target bacteria more effectively, preserving the therapeutic potential of MATE 2, and reducing the risk of resistance development. These findings emphasize the importance of conducting a preliminary evaluation of bacterial concentration to intervene with accurate phage concentration, thereby ensuring an effective biocontrol. On the other hand, *L. lactis* showed for the first time an antagonistic activity against *Xcc*, effectively inhibiting its growth on YPGA plates. This aligns with previous reports demonstrating the antibacterial effect of *L. lactis* on both Gram-negative and Gram-positive bacteria, strengthening its role as a possible biocontrol agent in agricultural practices (Settier-Ramírez et al., 2021; Sabri et al., 2024a). Subsequent *in vitro* tests revealed that nisin exerts bactericidal activity against *Xcc* cells, suggesting its involvement in the antagonistic effect of *L. lactis*. The TEM analyses showed that nisin induces severe structural changes in *Xcc* cells, including alterations in the outer membrane structure and cell cytoplasm, compromising the viability. This activity against *Xcc* can be added to the long list of nisin’s antimicrobial effects against pathogenic bacteria (Villarreal et al., 2024). Notably, the MATE 2-nisin combination resulted in over 76% reduction in *Xcc* growth after 72 h of treatment, whereas phage and nisin alone showed a 67 and 45% reduction, respectively, underscoring the enhanced effectiveness of the antimicrobial combination. Indeed, the synergistic effect of phage-nisin combination has been previously reported, demonstrating that it is based on the complementary action of phages, which specifically lyse bacterial cells, and of nisin, which disrupts resistance mechanisms and bacterial physiology, resulting in a significant increase in their individual actions (Rendueles et al., 2022). This is in addition to a possible role of phages in constraining bacterial defense mechanisms against bacteriocins (Backman et al., 2024), making their combination of practical importance in ensuring more efficient antimicrobial activity, along with a reduced possibility of bacterial resistance.

For biocontrol purposes, the stability and the safety of biocontrol agents are mandatory. In this context, MATE 2 has already demonstrated stability over a wide range of pH and temperatures, and its genome is devoid of genes encoding virulence factors, antibiotic resistance, toxins, or genes related to lysogenic action (Sabri et al., 2024b). Additionally, *L. lactis* has “generally recognized as safe” (GRAS) status and is found in a wide range of environments and a large variety of food products (de Lacerda et al., 2016). *L. lactis* strains have already been isolated from stems of *Eucalyptus*, from the interior of aquatic plants, and from the inner tissues and leaves of sugarcane (de Lacerda et al., 2016). Furthermore, Golomb and Marco (2015) showed that certain strains of *L. lactis* are well adapted for growth on plants. Likewise, in our *in-planta* experiments, MATE 2 and *L. lactis* successfully remained in broccoli leaves for 14 dpi and their persistent antibacterial activity against *Xcc* was confirmed, indicating their suitability in controlling black rot disease.

In the management of black rot disease in broccoli plants, preventive applications of MATE 2 and *L. lactis* were more effective in reducing disease symptoms than their curative applications. These findings align with those of a prior study, which demonstrated that applying phages as a preventative agent could reduce bacterial infections more effectively than post-challenge application (Ahmadi et al., 2016). The results also showed that MATE 2 was more effective than *L. lactis* in controlling black rot. This differential efficacy could be attributed to their behaviors and interactions within the host plant. Phages, unlike other types of antimicrobial compounds, their post-application levels will increase at the expense of bacterial host survival. Additionally, their replication within bacterial cells enables them to maintain proximity to *Xcc* throughout the plant, which may allow them to effectively track and lyse *Xcc* cells within the plant. Consistent with the *in-vitro* results, the combined application of MATE 2 and *L. lactis* preventively against *Xcc* resulted in increased effectiveness, successfully preventing black rot symptoms by 71% in broccoli plants, while their individual applications reduced disease by 64 and 38%, respectively. These findings validate the potential of MATE 2 and *L. lactis* to act synergistically and boost significantly their individual actions for enhanced black rot control. In addition to nisin production, *L. lactis* can produce various antimicrobial compounds such as organic acids, hydrogen peroxide, diacetyl, reuterin, acetaldehyde, acetoin, carbon dioxide, and other bacteriocins (Siroli et al., 2020), which may in our case be involved in the antibacterial activity against *Xcc* and may positively interact with MATE 2 against *Xcc* infections in plant tissues. Whether this is the case, is yet to be determined.

Beyond their antimicrobial activities, LAB and phages have also demonstrated synergistic benefits in enhancing plant production. Papaianni et al. (2020) highlighted the diverse potential advantages of phages in influencing plant metabolism and enhancing defense mechanisms. Their research underscores the role of phages in agricultural systems by potentially boosting plant resilience and response to stress factors. Furthermore, Jaffar et al. (2023) demonstrated the broad-spectrum activities of *L. lactis* and its bacteriocin nisin, which contribute positively to plant growth, soil health, and overall crop productivity. Additionally, Raman et al. (2022) reported that *L. lactis* significantly promoted the root and shoot length of rice, as well as cabbage growth and production. Integrating these properties, the combination of MATE 2 and *L. lactis* emerges as a pivotal strategy in integrated pest management of black rot disease. However, further research is needed to explore their potential applications in field conditions against *Xcc* and their effects on cruciferous vegetable production.

This study extends previous research on *Xf*, reaffirming the broad-spectrum of antibacterial efficacy of MATE 2 and nisin-producing *L. lactis* (Sabri et al., 2024a,b) and their suitability for being used in plant systems; thus, supporting their application in *Xf*-infected plants. These attributes, together with ease of production, make them a practical and cost-effective strategy for dealing with *Xf* and *Xcc* infections in plants. Such investigations will also be crucial to translating these promising results into viable solutions for farmers, thus contributing to more resilient and productive agricultural systems.

## Data Availability

The original contributions presented in the study are included in the article/supplementary material, further inquiries can be directed to the corresponding author.
